# In vitro and in vivo effects of geranylgeranyltransferase I inhibitor P61A6 on non-small cell lung cancer cells

**DOI:** 10.1186/1471-2407-13-198

**Published:** 2013-04-22

**Authors:** Drazen B Zimonjic, Lai N Chan, Veenu Tripathi, Jie Lu, Ohyun Kwon, Nicholas C Popescu, Douglas R Lowy, Fuyuhiko Tamanoi

**Affiliations:** 1 Molecular Cytogenetics Section, Lab. of Experimental Carcinogenesis, National Cancer Institute, National Institutes of Health, Bethesda, MD, USA; 2 Department of Microbio., Immunol. & Molec. Genet., Jonsson Comprehensive Cancer Center, University of California, Los Angeles, CA, USA; 3 Department of Chemistry and Biochemistry, University of California, Los Angeles, CA, USA; 4 Laboratory of Cellular Oncology, National Cancer Institute, National Institutes of Health, Bethesda, MD, USA

**Keywords:** Geranylgeranyltransferase I inhibitor, P61A6, Lung cancer, RhoA, Deleted in liver cancer 1

## Abstract

**Background:**

Lung cancer is the leading cause of cancer-related mortality. Therapies against non-small cell lung cancer (NSCLC) are particularly needed, as this type of cancer is relatively insensitive to chemotherapy and radiation therapy. We recently identified GGTI compounds that are designed to block geranylgeranylation and membrane association of signaling proteins including the Rho family G-proteins. One of the GGTIs is P61A6 which inhibits proliferation of human cancer cells, causes cell cycle effects with G1 accumulation and exhibits tumor-suppressing effects with human pancreatic cancer xenografts. In this paper, we investigated effects of P61A6 on non-small cell lung cancer (NSCLC) cells *in vitro* and *in vivo*.

**Methods:**

Three non-small cell lung cancer cell lines were used to test the ability of P61A6 to inhibit cell proliferation. Further characterization involved analyses of geranylgeranylation, membrane association and activation of RhoA, and anchorage-dependent and –independent growth, as well as cell cycle effects and examination of cell cycle regulators. We also generated stable cells expressing RhoA-F, which bypasses the geranylgeranylation requirement of wild type RhoA, and examined whether the proliferation inhibition by P61A6 is suppressed in these cells. Tumor xenografts of NSCLC cells growing in nude mice were also used to test P61A6’s tumor-suppressing ability.

**Results:**

P61A6 was shown to inhibit proliferation of NSCLC lines H358, H23 and H1507. Detailed analysis of P61A6 effects on H358 cells showed that P61A6 inhibited geranylgeranylation, membrane association of RhoA and caused G1 accumulation associated with decreased cyclin D1/2. The effects of P61A6 to inhibit proliferation could mainly be ascribed to RhoA, as expression of the RhoA-F geranylgeranylation bypass mutant rendered the cells resistant to inhibition by P61A6. We also found that P61A6 treatment of H358 tumor xenografts growing in nude mice reduced their growth as well as the membrane association of RhoA in the tumors.

**Conclusion:**

Thus, P61A6 inhibits proliferation of NSCLC cells and causes G1 accumulation associated with decreased cyclin D1/2. The result with the RhoA-F mutant suggests that the effect of P61A6 to inhibit proliferation is mainly through the inhibition of RhoA. P61A6 also shows efficacy to inhibit growth of xenograft tumor.

## Background

Attempts to inhibit tumor growth by blocking membrane association of signaling proteins have been pursued over the years [1]. One such method, inhibition of protein geranylgeranyltransferase type I (GGTase-I), has recently emerged as a promising anticancer approach [2,3]. Validation of GGTase-I as a target for anticancer drug development comes from studies using conditional knockout of the β-subunit of GGTase-I, which have indicated that genetic inactivation of GGTase-I reduced the growth of a K-ras-induced mouse lung tumor and increased survival [4]. GGTase-I catalyzes the geranylgeranylation of proteins containing the CAAL motif (C is cysteine, A is aliphatic amino acid and L is leucine) at their C-termini. Many of the proteins that are modified by GGTase-I are members of the Ras superfamily of GTPases, including RhoA, Rac, and Cdc42, which play important roles in human cancer [5-8]. It has been shown that slowed growth of mouse embryonic fibroblasts (MEFs) derived from cells defective in GGTase-I was reversed by expressing mutant forms of both RhoA and Cdc42 that can bypass the geranylgeranylation requirement [4], suggesting that the effects of GGTase-I inhibition are largely mediated by these Rho family proteins.

A variety of small molecule candidate inhibitors of GGTase-I (GGTIs) have been developed over the years. Peptidomimetic inhibitors based on the CAAL motif that is recognized by GGTase-I were the first class of GGTIs to be developed [9]. High throughput screening of a chemical compound library led to the identification of GGTI-DU40 [10]. Recently, we have described the development and characterization of novel small molecule GGTIs [11-14]. In our screen, we identified several GGTI compounds with novel scaffolds from a library constructed through phosphine-catalyzed annulation reactions, using allenoate as starting materials. These GGTIs specifically inhibit GGTase-I by competing with protein substrates. One of the GGTIs identified, P61A6, which has a dihydropyrrole ring as its core scaffold, showed inhibition of geranylgeranylation without affecting farnesylation and inhibited both proliferation and cell cycle progression in a variety of human cancer cell lines. P61A6, which has a remarkably long plasma half-life, also had significant tumor-suppressing effects with human pancreatic cancer xenografts [13].

Lung cancer is the leading cause of cancer-related mortality as seen from the estimated number of new cases and deaths from lung cancer (non-small cell and small cell combined) for 2012 in the US according to the National Cancer Institute; 226,160 and 160,340, respectively [15]. There are two groups of lung cancer. Non-small cell lung cancers (NSCLC) account for about 80% of cases, while small-cell lung cancers account for the remaining 20% [15]. We are particularly interested in non-small cell lung cancer (NSCLC), which is relatively insensitive both to chemotherapy and radiation therapy [16]. Another reason to focus on NSCLC is that one of the major tumor suppressor genes *DLC1*[17] is down-regulated or inactivated due to genetic and epigenetic mechanisms in a high proportion of primary NSCLC and derived cell lines [18,19]. *DLC1* encodes a GTPase activating protein (GAP) for Rho proteins [17,20,21], and loss of DLC1 expression in NSCLC cell lines is associated with increased RhoA-GTP [22,23].

In this paper, we address two preclinical issues. First, we show that GGTI P61A6 inhibits proliferation and transformed phenotypes of NSCLC cells, including the growth of xenograft tumors in mice. Second, we demonstrate the specificity of P61A6 by showing that a RhoA mutant whose biological activity is independent of GGTase-I renders the cells resistant to inhibition by P61A6.

## Methods

### Cell lines and cell cultures

NSCLC cell lines, H358, H23 and H1507, kindly provided by Dr. Curtis Harris (National Cancer Institute, Bethesda, MD), were maintained in RPMI 1640 medium (Cellgro, Herndon, VA). The medium was supplemented with 10% (v/v) fetal bovine serum (FBS; HyClone, Logan, UT) and 1% penicillin/1% streptomycin stock solution (Invitrogen, Carlsbad, CA). All cells were cultured at 37°C in a humidified incubator at 5% CO_2_.

### Compound

GGTI P61A6 was synthesized by coupling P5-H6 [14] with an L-phenylalanamide, where the free acid L-phenylalanine is converted to an amide. A 20 mM stock solution of P61A6 in DMSO was kept at −20°C until use.

### Cell proliferation and cell cycle analyses

Effects of P61A6 on cell proliferation were examined using the CCK-8 cell counting kit (Dojindo Molecular Technologies, Kumamoto, Japan) as described previously [14]. Briefly, cells (2.5 × 10^3^) were seeded onto 96-well plates. The following day, cells were treated with the appropriate inhibitor as indicated in the figure legends. The cell proliferation assay was performed in triplicate every other day. Data of each experimental series were tested against the controls (DMSO) for statistical significance, using Student’s paired two-tailed test. The cell cycle profiles were analyzed by flow cytometry (UCLA Flow Cytometry Core Facilities) as described previously [24].

### Western blotting

Cells were treated with DMSO or P61A6 for 48 h, harvested, and lysed in lysis buffer (1% Triton X-100, 150 mM NaCl, 20 mM Tris–HCl at pH 7.5, 1 mM EDTA, and 1× protease inhibitor mixture). Proteins were then resolved by 12% or 12.5% SDS-PAGE and immunoblotted with antibodies against p21^CIP1/WAF1^ (Millipore, Temecula, CA), p27^Kip1^ (rabbit, Santa Cruz Biotechnology, Inc.), RhoGDI (Santa Cruz Biotechnology, Inc.), RhoA (mouse, Santa Cruz Biotechnology), cyclin D1/2 (Millipore), the unprenylated form of Rap1 (U-Rap1; Santa cruz Biotechnology, Inc.), or actin (Calbiochem). Detection was performed using peroxidase-conjugated secondary antibodies (Biorad) and Amersham ECL Plus™ Western Blotting Detection Reagents (GE Healthcare Life Sciences). Select bands were quantified using ImageJ imaging processing program (National Institutes of Health).

### Subcellular fractionation

Cells were treated with DMSO or P61A6 for 48 h. Cells were then washed and scraped into PBS and centrifuged at 2,500 rpm for 5 min. Pellets were resuspended (10 mM HEPES/KOH at ph 7.3, 10 mM KCl, 5 mM MgCl_2_, 0.5 mM DTT, and 1× protease inhibitor mixture), incubated on ice for 30 min, and homogenized. Homogenates were centrifuged at 1000 × *g* for 10 min to collect the cytosolic fractions (supernatant). The remaining pellets were then resuspended in buffer containing 1% Triton X-100, 150 mM NaCl, 20 mM Tris–HCl at pH 7.5, 1 mM EDTA, and 1× protease inhibitor mixture, and centrifuged at 15,000 rpm for 15 min to collect the membrane-containing fractions (supernatant). Na^+^/K^+^ ATPase-α and RhoGDI or GAPDH were used as markers for the membrane-containing fractions and the cytosolic fractions, respectively.

### GTP-bound RhoA pull-down assay

Cells were serum-starved in the presence of DMSO or P61A6 for 24 h. Cells were then stimulated with 10% FBS in the presence of DMSO or P61A6 for 30 min. Whole cell lysates were collected using Mg^2+^-containing buffer, and GTP-RhoA was pulled down using GST-tagged Rhotekin-RBD protein beads (Cytoskeleton). Whole cell lysates (inputs for pull-down) and pull-down were resolved on SDS-PAGE for immunoblotting analysis, using RhoA antibodies (mouse, Santa Cruz Biotechnology) to detect total RhoA and GTP-bound-RhoA.

### Anchorage independent growth assay

Cells were seeded at a density of 20,000 cells/well in duplicate in 6-well culture dishes in 0.4% agar over a 0.8% bottom agar layer. Various concentrations of P61A6 or DMSO were added to the top layer of cells. Cultures were re-fed and treated with the GGTI or DMSO once weekly (14 days of incubation in total). Colonies were stained with 1 mg/ml MTT (tetrazolium salt) for 1 hour and scanned.

### Generation of stable H358 cells expressing RhoA-F

H358 cells were plated on 6-well plates and after 18 hours transfected with pcDNA3.1-3xHA-RhoA (wild-type, geranylgeranylated) and pcDNA3.1-3xHA-RhoA-F (farnesylated mutant) [12] using Lipofectamine™ 2000 (Invitrogen, Carlsbad, CA) according to manufacturers instructions. Construction of these plasmids has been described previously [12]. 10 μl of transfection reagent and 5.0 μg of plasmid DNA were diluted in 250 μl of OPTI-MEM medium (Invitrogen, Carlsbad, CA) and incubated at room temperature for 5 min. Both reagents and DNA were mixed and allowed to form complexes for 20 min at room temperature. The complexes were added to cells in 6-well plates that were 80% confluent, in serum-free RPMI medium without antibiotics, and incubated at 37°C for 6 hours. Medium was replaced with RPMI containing 10% FBS and antibiotics. Cells were further incubated for 48 hours. To generate stable cell clones, cells were trypsinized and plated at 1:10, 1:20, and 1:50 dilutions with selective medium containing 1000 μg/ml of Geneticin (Invitrogen, Carlsbad, CA). Stable clones were selected, expanded, and analyzed for expression of RhoA and RhoA-F by western blotting with anti-HA antibody.

### *In vivo* tumorigenicity; H358 xenografts in BALB/C nude mice

H358 cells were grown to 75% confluency, washed twice in PBS, and resuspended in DMEM/F12 media prior to injections. Twenty six week old female BALB/cAnNCr-nu/nu mice were obtained from NCI-Frederick Animal Production Program (Frederick, MD) and housed in an NCI animal facility. One mouse died one day after arrival, and the other nineteen were injected with 4x10^6^ H358 cells in 200 μL DMEM/F12 media. Ten animals received a single subcutaneous injection of the cells in the left sub scapular region, whereas the other nine on both sides. Three weeks after injection, all of the animals had palpable tumors that were 3–5 mm along their longer axis, and at that point both the unilaterally- and bilaterally-injected animals were randomly divided into experimental and control groups, with ten (5 + 5) and nine (4 + 5) animals, respectively. P61A6 (MW = 579.20) was dissolved in DMSO to make a 20 mM stock solution (24 mg of P61A6 in 2.071 mL DMSO, fin. conc. 11.589 mg/mL), which was aliquoted and stored at −20°C. Immediately before each treatment, the stock was diluted with 0.9% saline to make 160 μM injection solution of GGTI. Animals from the experimental group (individual weight ~20 g) were injected five times per week with up to 260 μM of this solution to provide a final dose of 1.2 mg/kg/treatment. Corresponding controls were injected with the appropriate volumes of 0.9% NaCl. Tumors were measured twice per week using a digital caliper, and tumor volumes were calculated using the following formula Tumor Volume = 4/3×3.14×(L/2xW/2×W/2), where L and W were the tumor length and width, respectively. Animals were sacrificed by cervical dislocation 48 days after being injected with H358 cells, and tumors were extirpated and compared for size. Randomly chosen samples from both control and treated group were used for histopathologic analysis and for assessing RhoA-GTP. The care and use of laboratory animals was in accordance with the principles and standards set forth in the Principles for Use of Animals (NIH Guide for Grants and Contracts), the Guide for the Care and Use of Laboratory Animals, the provisions of the Animal Welfare Acts, and all procedures were approved by National Cancer Institute Animal Care and Use Committees.

## Results

### P61A6 inhibits proliferation of non-small lung cancer cells

**Figure 1:**
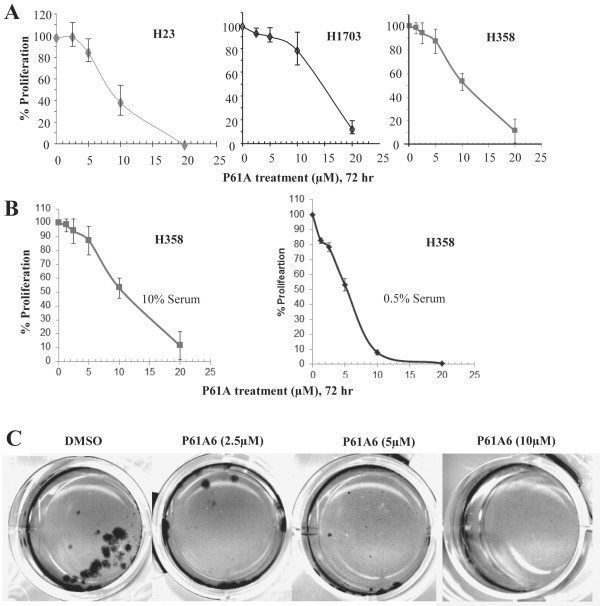
**Effects of P61A6 on growth of lung cancer cells. A**. H23, H1703 and H358 cells were treated with DMSO or the indicated concentrations of P61A6 for 72 hours and surviving cell numbers were counted with cell counting kit, normalized to the control cells without treatment. **B**. H358 cells under normal growth conditions (medium with 10% FBS) or nutrient-starved conditions (medium with 0.5% FBS) were treated with GGTI P61A6 for 72 hours and cell proliferation relative to the DMSO control (100%) is plotted. **C**. H358 cells were seeded at a cell density of 20,000 cells/well in 6-well culture dishes in 0.4% agar over a 0.8% bottom agar layer. Various concentrations of P61A6 or DMSO were included in the top layer of cells. Cultures were re-fed and treated with P61A6 or DMSO once weekly (14 days of incubation in total). Colonies were stained with 1 mg/ml MTT for 1 hour and scanned. This experiment was carried out twice with similar results.

Effects of P61A6 on the proliferation of non-small cell lung cancer (NSCLC) cells as monolayer cultures were examined using three different cell lines, H358, H23 and H1507. As shown in Figure 1A, proliferation of each line was inhibited by P61A6 in a dose-dependent manner, with an IC50 ranging from 5 to 15 μM. The sensitivity of H358 cells to P61A6 was further increased when the cells were grown under nutrient-starved conditions (in 0.5% serum) (Figure 1B). When inhibition of anchorage-independent growth of H358 was examined by soft agar assay, which is more stringent than monolayer growth, P61A6 induced substantial growth inhibition at concentrations as low as 5 μM (Figure 1C). In the subsequent experiments, we focused on H358, whose sensitivity to P61A6 was between that of H23 and H1703.

**Figure 2:**
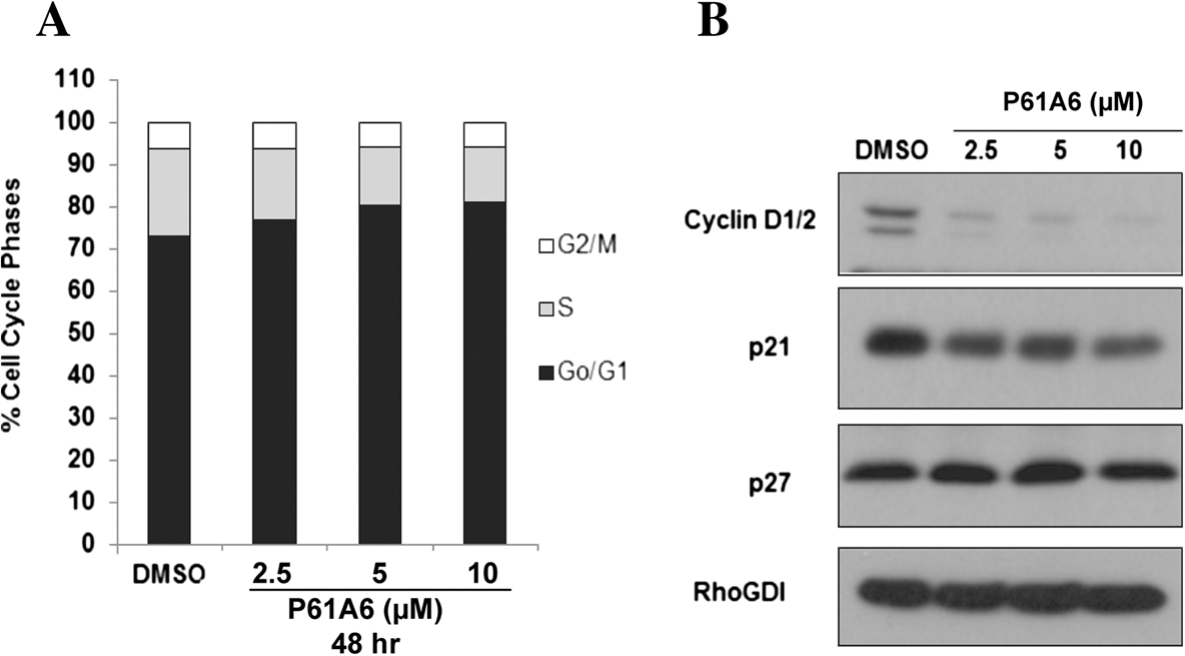
**Effects of P61A6 on cell cycle progression. A**. H358 cells were treated with DMSO or P61A6 for 48 hours. Cell cycle profiles were monitored by flow cytometry. The percentages of cells in each phase of the cell cycle are indicated by different shades. Shown are representative results from three independent experiments. **B**. Whole cell lysates from cells treated with DMSO or P61A6 for 48 hours were prepared and resolved on SDS-PAGE for immunoblotting using antibodies against cyclin D1/2, p21^CIP1/WAF1^, p27^Kip1^, or RhoGDI (loading control). Data shown are representative of two independent experiments.

To examine possible cell cycle effects of P61A6, H358 cells were treated with varying concentrations of P61A6, and then cell cycle was analyzed by flow cytometry. The results after 48 hours of treatment are shown in Figure 2A. The percentage of G0/G1 phase cells increased. This increase was associated with a concomitant decrease in the percentage of S phase cells, while the percentage of G2/M phase cells did not change. To investigate the effect of P61A6 further, we examined effects on cell cycle regulators involved in the G1/S transition, namely cyclin D1/2, p21^CIP1/WAF1^, and p27^Kip1^. As shown in Figure 2B, P61A6 caused a significant decrease in cyclinD1/2. On the other hand, the levels of Cdk inhibitors p21^CIP1/WAF1^ and p27^Kip1^ were less affected by P61A6.

### P61A6 inhibits protein geranylgeranylation and activation of RhoA, and its anti-proliferative effects are mainly attributable to RhoA

**Figure 3:**
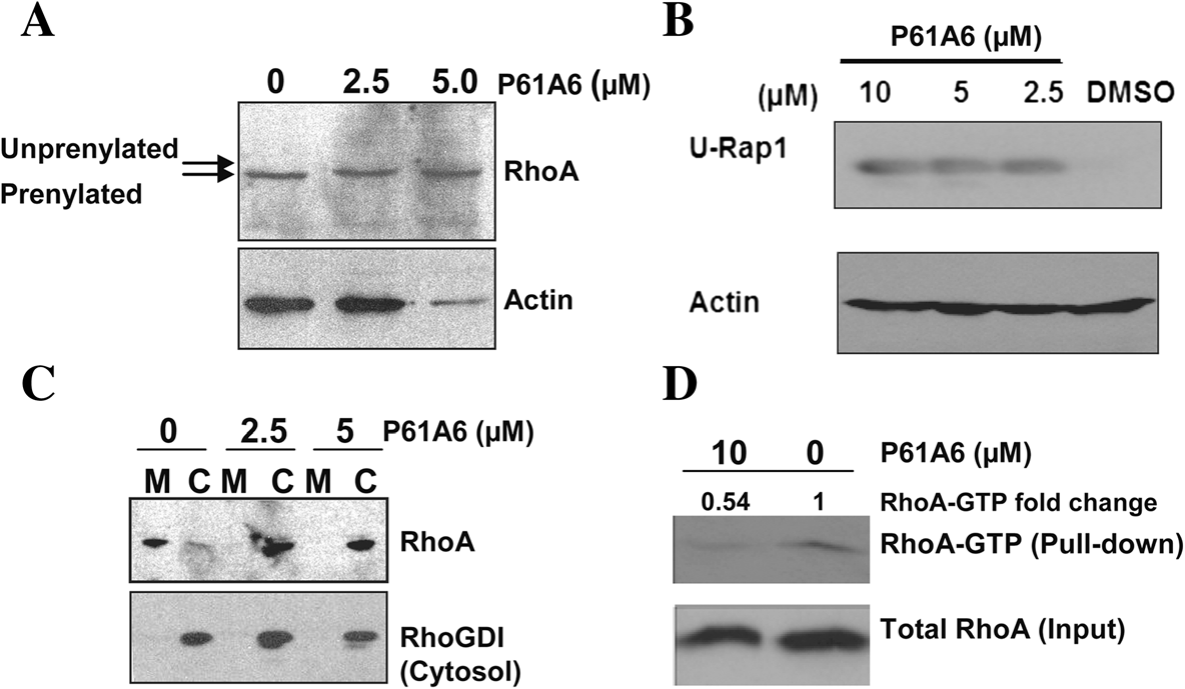
**P61A6 inhibits protein geranylgeranylation and activation of RhoA. A**. Effects of P61A6 on geranylgeranylation of RhoA. H358 cells were treated with DMSO or increasing concentrations of P61A6 for 48 h. Whole cell lysates were prepared and analyzed for RhoA (upper panel) and actin (lower panel). **B**. Appearance of unprenylated form of Rap1 by treating the cells with P61A6. Rap1 is normally geranylgeranylated but the P61A6 treatment produces unprenylated Rap1 and this is detected by using an antibody specific for unprenylated Rap1. **C**. P61A6 causes an increase in cytosolic RhoA. H358 cells were treated with DMSO or P61A6 for 48 h. Cytosolic (C) and membrane (M) fractions were prepared and processed for SDS-PAGE, followed by Western blotting. Upper panel: RhoA; lower panel: cytosol marker RhoGDI. **D**. P61A6 inhibits RhoA activation in H358 cells. Cells were serum-starved in the presence of DMSO or P61A6 for 24 h. Then, cells were stimulated with 10% FBS in DMEM in the presence of DMSO or P61A6 for 30 min. Whole cell lysates were collected using Mg^2+^-containing lysis buffer, and GTP-RhoA was pulled down using GST-tagged Rhotekin-RBD protein beads (Cytoskeleton) following the manufacturer’s instructions. Whole cell lysates (inputs) and pull-down were resolved on SDS-PAGE for immunoblotting analysis using RhoA antibodies to detect total RhoA (bottom panel) and GTP-bound-RhoA (top panel), respectively.

To investigate the mechanism of P61A6 effects, we focused on the GTPase RhoA, which has emerged as a major effector of GGTase-I deficiency in previous studies [4,12]. Also, H358 cells do not express detectable levels of the RhoGAP DLC1 and have high RhoGTP levels [23]. Figure 3A shows that P61A6 inhibited geranylgeranylation of RhoA, as detected by the upper mobility shift of the RhoA band due to the inhibition of prenylation. In addition, treatment of cells with P61A6 led to the appearance of the unprenylated form of Rap1 (Figure 3B), implying that protein geranylgeranylation in general is inhibited in these cells. To examine whether P61A6 inhibits membrane association of RhoA, we separated whole cell extracts into membrane and cytosolic fractions, and examined the amount of RhoA in each fraction. As shown in Figure 3C, most RhoA was detected in the membrane fraction in the control DMSO treated cells. However, after treatment with P61A6, the amount of RhoA in the membrane fraction decreased dramatically, and RhoA became predominantly cytosolic. Finally, we examined whether P61A6 inhibits serum-dependent activation of RhoA (Figure 3D). Serum-starved H358 cells were stimulated by the addition of serum, and the amount of GTP-loaded RhoA was assessed by GST-tagged Rhotekin-RBD beads. Treatment with P61A6 significantly decreased the amount of Rho-GTP pulled down, whereas the total amount of RhoA was unaffected by the treatment. Taken together, these results show that P61A6 has significant effects on RhoA.

**Figure 4:**
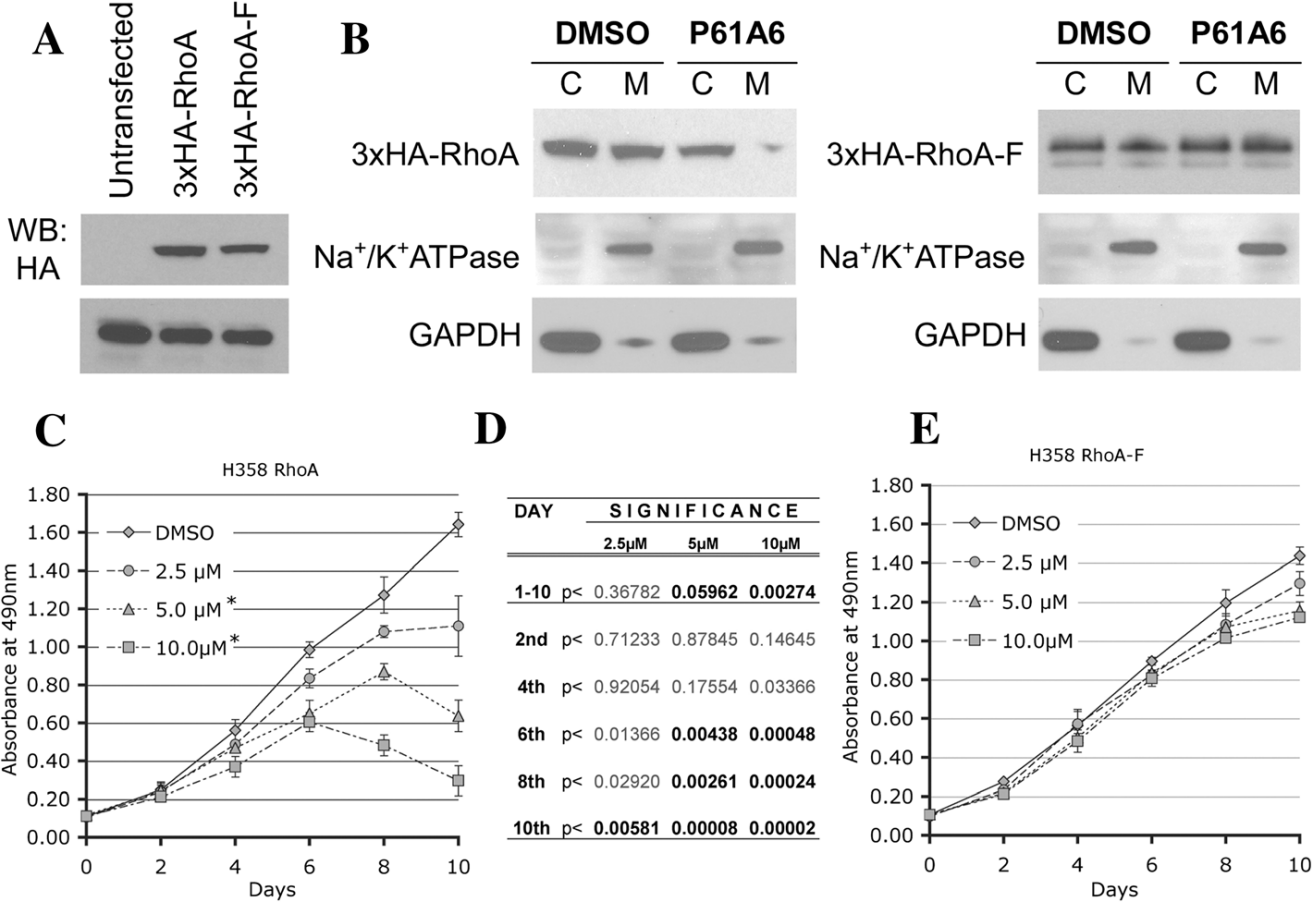
**Expression of RhoA-F suppresses the ability of P61A6 to inhibit proliferation. A**. Western blotting with anti-HA antibody showing expression of stably transfected 3xHA-RhoA and 3xHA-RhoA-F constructs in H358 cells.** B**. Stably transfected H358 cells were treated with DMSO or 10 μM P61A6 for 48 hours. Membrane and cytosolic fractions were prepared and examined for RhoA (Left Panel) and RhoA-F (Right Panel) localization on membrane and cytosolic fractions by Western blotting. Na+/K + ATPase and GAPDH were used as control for membrane and cytosolic fractions, respectively. **C**. H358 RhoA and **E**. H358 RhoA-F cells were treated with DMSO or P61A6 at different concentrations (2.5 μM, 5.0 μM and 10 μM) under low serum conditions (medium with 0.5% FBS) for 10 days. Cell proliferation assay was performed in triplicate every 2nd day for 10 days. **D**. Differences in proliferation rate between cells treated with different concentrations of P61A6 and DMSO-treated control is characterized by increasing statistical significance.

As described above, P61A6 induces decreased levels of cyclin D1 together with increased G1 and decreased proliferation. A number of studies in lung cancer cells suggest that RhoA plays important roles in cyclin D1 and cell cycle progression [25,26]. To rigorously test the hypothesis that RhoA is a key target of the growth inhibitory effects of P61A6, we transfected H358 cells with the wild type RhoA (3xHA-RhoA) or a mutant form of RhoA, RhoA-F (3xHA-RhoA-F), which can be farnesylated instead of geranylgeranylated, because the C-terminal leucine has been changed to serine. Clones stably expressing either wild type RhoA or the RhoA-F mutant were established. When these clones were tested with anti-HA antibody, a similar level of expression was observed for both proteins (Figure 4A). Geranylgeranylation of RhoA and farnesylation of RhoA-F expressed from these constructs have been confirmed previously [12]. To compare the sensitivity of these clones to P61A6-induced inhibition of RhoA membrane association, we treated the clones with DMSO or 10 μM P61A6 for 48 hours, and membrane and cytosolic fractions were prepared and immunoblotted with anti-HA antibody for transfected RhoA and RhoA-F localization. Treatment with P61A6 inhibited membrane association of wild type RhoA, as shown by its disappearance from the membrane fraction (Figure 4B, Left Panel), but there was no change in the level of RhoA-F in the membrane fraction (Figure 4B, Right Panel), showing for this parameter that RhoA-F was resistant to P61A6 treatment. To assess the effects of P61A6 on cell proliferation, H358 RhoA (Figure 4C) and H358 RhoA-F (Figure 4E) cells were treated with DMSO or P61A6 at various concentrations under low serum (0.5% FBS) conditions for 10 days. Proliferation of P61A6-treated RhoA-F cells was not significantly different from the controls, whereas the treatment of RhoA cells with P61A6 significantly inhibited cell proliferation compared to DMSO-treated controls (Figure 4D,E). These results confirm that P61A6 inhibits geranylgeranylation but not farnesylation and, importantly, indicate that the vast majority of the growth inhibitory effects of P61A6 on the cells depend upon the inhibition of RhoA by P61A6.

### P61A6 inhibits growth of H358 xenograft tumor in mice

**Figure 5:**
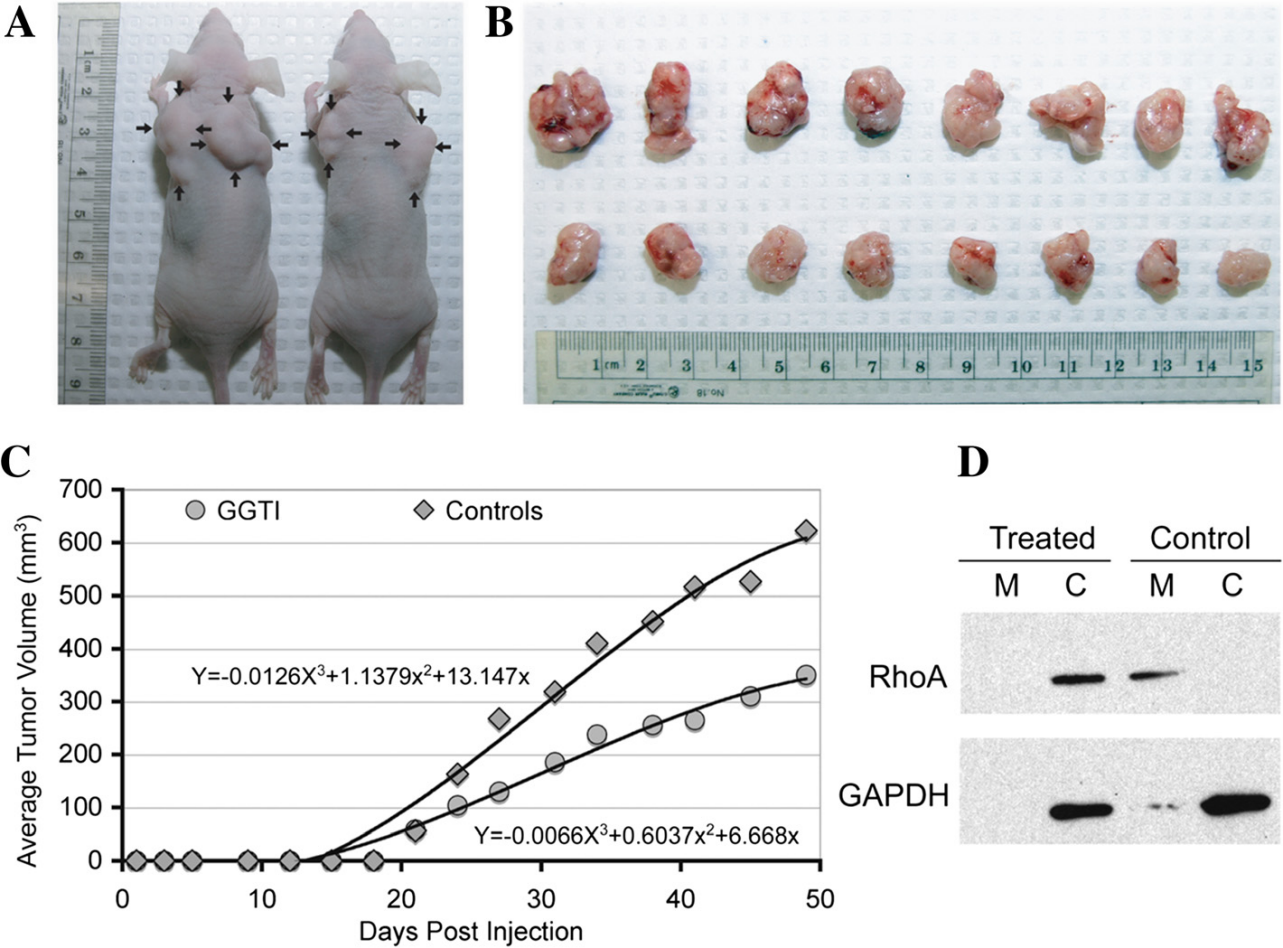
**Effect of P61A6 on tumorigenicity of H358 cells in vivo and intracellular distribution of RhoA in H358 xenografts.** The treatment was started 3 weeks after subcutaneous inoculation of the cells when palpable tumors reached 3–5 mm in diameter. 5 times/week i.p. injections were performed until the end of experiment. **A**. Animals injected with P61A6 (right) exhibited smaller tumor compared to controls in situ (left). **B**. Comparison of the eight largest extirpated tumors from control animals (top) and the eight largest from animals treated with P61A6 (bottom) demonstrates the effects of treatment on xenograft tumor growth. **C**. The average tumor volumes of P61A6-treated and untreated groups is shown. **D**. Protein extracted from cytosolic and membrane fractions of tumors from P61A6-treated and untreated animals was western blotted for RhoA to evaluate the RhoA association with membrane. (M, membrane fraction; C, cytosolic fraction).

H358 tumor xenografts were established in nude mice as described in the Method section. The maximum tolerated dose and toxicity of GGTI P61A6 were determined in previous experiment [13]. In that study, P61A6 ranging from 0 to 4.64 mg/kg was used. While we did not observe any significant toxicity, a slight hepatoxicity was detected in mice treated with the two highest doses. Therefore, 1.12 mg/kg P61A6 was chosen for the present experiment. The treatment with P61A6 was started 3 weeks after subcutaneous inoculation of the cells, when the tumors reached 3–5 mm in diameter and were palpable. 5 times/week i.p. injections were performed until the end of experiment. Mice inoculated with H358 cells and treated with P61A6 exhibited visibly smaller tumors in situ (Figure 5A), and comparison of the largest extirpated tumors from both P61A6-treated and control animals confirmed that difference (Figure 5B). In both the control and the treated groups, we observed a few satellite tumors, which developed near the main ones and appeared to have resulted from local invasion. Comparison of average tumor volumes between control and P61A6-treated groups (Figure 5C) indicated the degree to which tumor growth was inhibited by P61A6 treatment. In eight out of nine successive measurements, the difference in average tumor volume between two groups was statistically significant (Student’s t-test), with the p value < 0.01 on 25^th^ day of the experiment and p < 0.008 on the last, 48^th^, day. In tumors from the controls and P61A6-treated animals, we checked for the intracellular distribution of RhoA protein as an indicator of geranylgeranylation inhibition. Analysis of cell membrane and cytosolic fractions of tumors probed for RhoA showed (Figure 5D) that the RhoA protein is mostly confined to cytoplasm in the P61A6-treated group, in sharp contrast to control animals, where the protein is almost exclusively associated with membranes, demonstrating that GGTI treatment has effectively inhibited the prenylation required for effective membrane association of RhoA.

## Discussion

In this paper, we have shown that P61A6 (GGTI) has significant anti-tumor effects on NSCLC cells *in vitro* and *in vivo*. Detailed analyses of the effects of P61A6 on one of the NSCLC cell lines, H358, showed that P61A6 inhibited anchorage-dependent and -independent growth of the cells, caused cell cycle effects, and inhibited the growth of mouse xenograft tumors whose treatment was initiated after the tumors became palpable. In GGTI-treated tumors, membrane association of RhoA was dramatically reduced, consistent with the presumed mechanism of action of P61A6. Since our previous P61A6 studies have focused on pancreatic cancer, this paper provides the first evidence to suggest that P61A6 may suppress tumorigenecity of NSCLC.

Another important contribution of this paper concerns the mechanism of action of P61-A6 on NSCLC cells, by providing evidence that RhoA plays critical roles in the effects of P61A6 on H358 cells. First, we have demonstrated that P61A6 inhibits geranylgeranylation as well as membrane association of RhoA, which is known to be geranylgeranylation-dependent. Consistent with this result, activation of RhoA - examined by determining the serum response to serum-starved cells - was blocked by the treatment with P61A6. In addition, we have shown that expression of a mutant form of RhoA (Rho-F) that can bypass the geranylgeranylation requirement abrogates the inhibition of RhoA membrane assocation and the inhibition of proliferation by P61A6. While other proteins such as Rac, Ral and RhoB have previously been suggested to play a role in GGTI effects in other cell lines [27-29], our study suggests that the effects of P61A6 on H358 lung cancer cells are largely mediated by RhoA.

Further characterization provided an overall view of the action of P61A6. We found that P61A6 induces accumulation of G1 phase cells, one of the hallmarks of GGTI effects [30], and that the level of cyclin D1/2 was decreased by P61A6 treatment. The significance of cyclin D1 in tumor growth and metastasis of NSCLC cells has been shown by the use of cyclin D1-targeted siRNA [31]. In addition, RhoA has been shown to play critical roles in cyclin D1 expression, cell cycle, and proliferation of lung cells [25,26]. Together with our demonstration that RhoA plays a major role in the effects of P61A6, the general scheme for the action of P61A6 on H358 may be summarized in the following way: P61A6 inhibits RhoA, leading to a decrease in cyclin D1/2, which results in G1 cell cycle arrest and inhibition of proliferation. There could, however, be variations to this general idea. In H358 cells, we have shown that P61A6 affects cyclin D1/2, while the levels of Cdk inhibitors p21^CIP1/WAF1^ and p27^Kip1^ are not significantly affected. In other cell lines, such Panc-1, however, we have observed increased p21^CIP1/WAF1^ levels after GGTI treatment [12,14]. The differences might be attributable to divergence in the levels of these cell cycle regulators in different cell lines. In fact, we noted that, in contrast to cyclin D1/2, the levels of p21^CIP1/WAF1^ and p27^Kip1^ are quite high in H358 even before treatment, which may have contributed to P61A6 having a more pronounced effect on cyclin D1/2 than on p21^CIP1/WAF1^ or p27^Kip1^.

One issue that requires further investigation concerns effects of GGTI on RhoA activation. In our experiment, we showed that the activation of RhoA in response to serum stimulation is blocked by GGTI in lung cancer cells. This is consistent with other studies in endothelial and breast cancer cells. In endothelial cells, GGTI-286 blocked increase of RhoA-GTP induced by monocyte adhesion [32]. GGTI-286 also blocked GTP-loading of RhoA induced by thrombin in endothelial cells [33]. In breast cancer cells, RhoA activity as detected by RhoA-GTP was inhibited by GGTI-298 [34]. However, Khan et al. [35,36] reported that GGTase-I deficiency in macrophage resulted in the accumulation of RhoA-GTP. Further studies are needed to examine how GGTase-I deficiency influences RhoA activation in different cellular contexts.

Down-regulation and inactivation of DLC1 expression through genetic and epigenetic alterations in multiple malignancies may represent the most frequent mechanism for aberrant activation of Rho GTPases in human oncogenesis [37]. Activity of Rho GTPases is elevated in many human cancers and their metastases, and the oncosuppressive effect of DLC1 requires RhoGAP activity, which negatively regulates Rho GTPases, most commonly RhoA [5,38]. The observation that down-regulation of DLC1 in NSCLC is associated with a poor clinical outcome [39] implies that targeting pro-oncogenic pathways activated by this down-regulation could be especially useful therapeutically, and inhibition of the RhoA pathway and Rho kinase, a downstream effector of Rho, are promising options for therapeutic interventions.

## Conclusions

Taken together, the present study clearly demonstrates that our novel GGTI P61A6 inhibits proliferation of NSCLC cells and causes G1 accumulation associated with decreased cyclin D1/2. The result with the RhoA-F mutant suggests that the effect of P61A6 to inhibit proliferation is mainly through the inhibition of RhoA. P61A6 also shows efficacy to inhibit growth of xenograft tumor. These results provide evidence that our GGTI P61A6 is a promising drug candidate for NSCLC therapy.

## Abbreviations

GGTase-I: Geranylgeranyltransferase type I; MEFs: Mouse embryonic fibroblasts; GGTIs: Inhibitors of GGTase-I; NSCLC: Non-small cell lung cancer; SCLC: Small cell lung cancer; GAP: GTPase activating protein.

## Competing interest

The authors declare that they have no competing interests.

## Authors’ contributions

DZ, LC, VT and JL carried out the cell experiments and drafted the manuscript. OK carried out the synthesis of compound GGTI P61A6 and helped to draft the manuscript. NP, DL, and FT participated in the design of the study and coordination and helped to draft the manuscript. All authors read and approved the final manuscript.

## Pre-publication history

The pre-publication history for this paper can be accessed here:

http://www.biomedcentral.com/1471-2407/13/198/prepub
